# Performance of Large Language Models in Oral Cancer Patient Education: An Evaluation of Reliability, Readability, and Patient Communication Quality

**DOI:** 10.3290/j.ohpd.c_2786

**Published:** 2026-08-26

**Authors:** Bo Zhang, Weiwei Shi, Ying Zhang

**Affiliations:** a Bo Zhang Supervising Nurse, Centre of Stomatology, China-Japan Friendship Hospital, Beijing, China; involved in the conceptualisation of the study, methodology, software, data curation, investigation, formal analysis, visualisation, and preparation of the original draft; b Weiwei Shi Associate Chief Nurse, Centre of Stomatology, China-Japan Friendship Hospital, Beijing, China; involved in the conceptualisation of the study, data curation, formal analysis, and supervision; c Ying Zhang Associate Chief Nurse, Centre of Stomatology, China-Japan Friendship Hospital, Beijing, China; involved in the formal analysis, supervision, resources, and review and editing of the manuscript

**Keywords:** AI; chatbot; health information; oral cancer; readability; reliability

## Abstract

**Background:**

As society is increasingly depending on large language models (LLMs) for health-related questions, it is essential to objectively evaluate the quality and accessibility of the oral cancer information they provide. Although LLMs occupy a growing space in digital health communication, it remains unknown whether the information they generate is both reliable and easy to read.

**Objective:**

The purpose of this study was to evaluate the reliability and readability, respectively, of the responses generated by four mainstream LLMs (ChatGPT, Gemini, Perplexity, and DeepSeek) to questions related to oral cancer. Specifically, the present authors aimed to evaluate the reliability of responses to common oral cancer questions and assess whether the readability of responses meets established expectations.

**Methods:**

Twenty-two commonly asked, patient-orientated oral cancer–related questions were developed through two predefined phases: Google Trends analysis and expert consultation with specialists in oral oncology. Each question was entered as an independent single-turn prompt into four LLMs: ChatGPT-5, Gemini 2.5, Perplexity Pro, and DeepSeek v3.2. The primary outcome was information reliability and quality, assessed using four standardized instruments: the DISCERN questionnaire, the Ensuring Quality Information for Patients (EQIP) tool, the Journal of the American Medical Association (JAMA) benchmark criteria, and the Global Quality Scale (GQS). The secondary outcome was readability, assessed using six established indices: the Automated Readability Index, Flesch Reading Ease Score, Gunning Fog Index, Flesch-Kincaid Grade Level, Coleman-Liau Index, and Simple Measure of Gobbledygook.

**Results:**

Significant differences were observed among the four LLMs in DISCERN, EQIP, and JAMA scores (all *P* < 0.001), whereas no significant difference was found in GQS scores (*P* = 0.440). Perplexity Pro achieved the highest mean DISCERN score (46.36 ± 4.70), EQIP score (85.00 ± 0.00), GQS score (4.05 ± 0.58), and JAMA score (1.00 ± 0.00). However, all models produced responses above the recommended sixth-grade readability level. The mean FKGL scores ranged from 12.65 ± 3.07 for ChatGPT-5 to 15.65 ± 3.36 for Perplexity Pro, and the mean FRES scores ranged from 37.50 ± 14.27 for Perplexity Pro to 52.59 ± 12.47 for Gemini 2.5.

**Conclusion:**

Current LLMs may support oral cancer patient education, but their use remains limited by variable information quality, insufficient transparency, and poor readability. Although Perplexity Pro performed better on several reliability-related metrics, no model showed consistently high performance across all dimensions or met recommended readability standards. Future LLM-based patient education tools should prioritise verifiable sourcing, guideline-based accuracy, risk communication, and plain-language adaptation.

Oral cancer is a type of head and neck cancer that arises in the lips, tongue, buccal mucosa, gingiva, floor of the mouth, hard palate, and retromolar trigone, with squamous cell carcinoma being the most common histological type.^18,27
^ Its epidemiology varies substantially across geographic regions and is strongly associated with lifestyle-related risk factors, particularly tobacco and alcohol consumption.^24^ According to global burden of disease data, oral cancer continues to cause hundreds of thousands of new cases and deaths each year, with substantial age-standardised incidence and mortality rates worldwide.^22^ Although the oral cavity is anatomically accessible for inspection, many patients are still diagnosed at an advanced stage, contributing to an overall 5-year survival rate that has historically remained approximately 50% to 60%.^19^ In contrast, survival can exceed 80% when oral cancer is detected and treated early.^16^ These marked prognostic differences between early and late diagnosis highlight the importance of accurate, understandable, and patient-orientated health information.

The public increasingly relies on the internet as a primary source of health information. However, the online health-related information is often characterised by inconsistent quality and unverified content and is potentially misleading, creating a gap between information availability and professional verification.^30^ For patients and the general public, this may result in the coexistence of information overload and practical knowledge scarcity. Therefore, reliable and readable patient education materials are essential for supporting oral cancer awareness, early symptom recognition, risk reduction, timely consultation, and informed communication with health care professionals.

Recent advances in generative AI and large language models (LLMs) have changed the way health information can be accessed. Members of the public can now obtain immediate, natural-language responses from AI-based systems regarding disease symptoms, prevention, diagnosis, treatment, and follow-up, which may lower the threshold for accessing health information.^13^ However, in serious medical contexts such as oral cancer, the reliability of LLM-generated information is a fundamental concern. Responses may be incomplete, outdated, unsupported by verifiable sources, or insufficiently explicit about risk warnings and the need for professional consultation.^11^ Readability is equally important. Even factually correct information may have limited educational value if it contains complex terminology or sentence structures that exceed the comprehension level of lay users.^23^ Several major US health care organisations, including the American Medical Association (AMA), the National Institutes of Health (NIH), and the Department of Health and Human Services, recommend that patient education materials should be written at or below a sixth-grade reading level to maximise accessibility.^26^ This requirement is particularly relevant for oral cancer education because understanding warning signs, risk factors, treatment options, and follow-up recommendations may influence health-seeking behaviour and patient–clinician communication. Nevertheless, systematic evaluations of AI-generated information in speciality medical fields, particularly oral cancer patient education, remain limited.^2^


Existing research on LLMs in health care has mainly focused on general medical information, clinical decision support, or selected disease areas, while less attention has been paid to whether mainstream LLMs can generate oral cancer information that is both reliable and readable for patient communication.^4^ Moreover, previous evaluations have often emphasised general response quality, whereas fewer studies have simultaneously assessed information reliability, transparency, and readability using standardised instruments. This represents an important evidence gap, because patient-facing oral cancer information should be not only medically trustworthy but also understandable to users with diverse levels of health literacy. Thus, the present authors conducted a multidimensional evaluation of four mainstream LLMs in responding to frequently asked oral cancer–related questions. The primary hypothesis was that the evaluated LLMs would differ in instrument-based reliability and information quality, and the secondary hypothesis was that the initial responses generated by these LLMs would exceed the recommended sixth-grade readability level for patient education materials. By combining quantitative readability assessment with expert-based evaluation of information quality, this study aimed to clarify the strengths and limitations of current LLMs in oral cancer patient education and to provide evidence for clinicians, patients, and developers seeking to improve AI-supported health communication.^28^


## Methods

### Study Design and Overview

The present authors conducted a cross-sectional appraisal of existing LLMs to assess the calibre of responses to questions related to oral cancer from the public perspective. The workflow consisted of provision of standard terminology, regimens of questions grounded in real-world information needs from prospective patients and input from experts, collection of model outputs under controlled conditions, and evaluation of readability and trustworthiness using standardised instruments.

### Standardised Terms and Question Development

To ensure consistency of terminology, standardised entry terms for ‘oral cancer’ were identified by consulting the Medical Subject Headings (MeSH) database. These terms included ‘cancer of mouth’, ‘cancer of the mouth’, ‘mouth cancer’, ‘neoplasms’, ‘mouth’, ‘oral’, ‘oral cancer’, and ‘oral, neoplasms’. These standardised terms informed the identification of relevant topics and the development of patient-orientated prompts.

Public information needs were characterised using Google Trends, which groups semantically related search terms across languages under a single ‘oral cancer’ topic and therefore provides an overview of global search interest.^13^ Search data from 2020 to 2025 were reviewed and 11 unique, highly related public queries were identified after removing duplicate or near-duplicate items. Because search data alone may not capture clinically important but less frequently searched issues, 11 additional questions were developed with input from oral oncology specialists. These expert-derived questions covered topics such as staging, prognosis, postoperative reconstruction, psychological support, and follow-up monitoring.

The final evaluation set comprised 22 patient-orientated questions for analysis. To improve thematic adequacy, these questions were mapped into six domains: general knowledge, symptoms and early detection, risk factors and prevention, diagnosis and staging, treatment and surgery, and prognosis, rehabilitation, and follow-up. This approach was intended to balance real-world public search interest with clinical relevance across the oral cancer care continuum. The present authors do not claim that these 22 questions exhaustively represent all possible oral cancer information needs; rather, they provide a focused, reproducible, and clinically relevant sample suitable for cross-model comparison. The complete list of 22 prompts is shown in Table 1. Additional information on prompt source, thematic domain, clinical relevance, and inclusion rationale is available from the authors upon request . All 22 prompts were entered verbatim into each AI system, and the original chatbot responses are also available from the authors upon request.

**Table 1 Table1:** The 22 oral cancer-related prompts used for chatbot evaluation

Question number	Prompt
1	Oral cancer symptoms
2	What is oral cancer
3	Tongue cancer
4	Oral cancer treatment
5	The population prone to oral cancer
6	Oral cancer causes
7	Oral cancer screening
8	HPV
9	HPV oral cancer
10	Oral cancer surgery
11	Gum cancer
12	Five-year survival rate for oral cancer
13	Prevention of oral cancer
14	Staging of oral cancer
15	The pathogenesis of oral cancer
16	Carcinoma of buccal mucosa
17	Carcinoma of mouth floor
18	Hard palate cancer
19	Lip cancer
20	Psychological guidance for patients with oral cancer
21	Tissue reconstruction after oral cancer surgery
22	Health monitoring and follow-up after oral cancer surgery


### Evaluation of AI Systems and Response Collection

Four AI systems were evaluated: ChatGPT-5, Gemini 2.5, Perplexity Pro, and DeepSeek v3.2. All prompts were entered as independent single-turn queries. To reduce potential bias from personalisation or previous conversational context, browsing cache and history were cleared before each query where possible. No follow-up prompts were used. This procedure was intended to capture the publicly accessible, user-facing behaviour of the models at the time of evaluation. Additional details on model information, access mode, context control, and response collection conditions are available from the authors upon request.

### Outcome Variables and Assessment Instruments

The primary outcome was instrument-based reliability and information quality of the LLM-generated responses. This outcome was assessed using the Determine Information Suitability, Content Reliability and Narrative quality (DISCERN) questionnaire, the Ensuring Quality Information for Patients (EQIP) tool, the Global Quality Scale (GQS), and the Journal of the American Medical Association (JAMA) benchmark criteria. The secondary outcome was readability, which was evaluated using six established indices: the Automated Readability Index (ARI), Flesch Reading Ease Score (FRES), Gunning Fog Index (FOG), Flesch-Kincaid Grade Level (FKGL), Coleman-Liau Index (CL), and Simple Measure of Gobbledygook (SMOG). Readability was assessed against the commonly recommended sixth-grade target for patient education materials.

### Readability Assessment

Readability was measured using six well-known formulas which were calculated using a professional online calculator (https://readabilityformulas.com), as follows:

ARI:^21^ (4.71 * (characters/words)) + (0.5 * (words/sentences)) − 21.43; FRES:^12^ (206.835 − (1.015 * (words/sentences)) − (84.6 * (syllables/words)); FOG:^12^ 0.4 * ((words/sentences) + (percentage of complex words)); FKGL:^12^ score = (0.39 x average sentence length [ASL]) + (11.8 x average syllables per word [ASW]) − 15.59; CL:^7^ score = 5.89(characters/words) − 0.3(sentences/words) − 15.8; SMOG:^10^ [square root](number of polysyllabic words) + 3. 

Consistent with the AMA and US NIH, the present authors compared the results to a sixth-grade target grade level.^11^ The passing threshold was established beforehand as FRES ≥ 80.0, and < grade six for each of the other five grade level formulas. The formulas, thresholds, and operational interpretation of the readability indices are available from the authors upon request.

### Reliability and Quality Appraisal

The reliability of the AI-generated information and general quality were evaluated with four tools:

DISCERN: This evaluates the quality of consumer health information, with a focus on treatment choices.^6^ Since the official manual does not provide a definitive interpretation of total scores and there are no universally agreed cut-points, the present authors adopted the depth classification criteria commonly used in existing literature, with grades defined as 63 to 75, excellent; 51 to 62, good; 39 to 50, fair; 27 to 38, poor; and 16 to 26, very poor.^25^
Ensuring Quality Information for Patients (EQIP): This assesses the quality of written patient information materials, broadly interpreting clarity and textual quality.^17^ The tool incorporates 20 items with four response options (‘yes’, ‘partly’, ‘no’, and ‘not applicable’). Scoring for the items was 1 for ‘yes’ 0.5 for ‘partly’, and 0 for ‘no’, with items marked as ‘not applicable’ excluded from the denominator. The percentage score was devised for each assessed document to be a calculation of [(sum of item scores ÷ number of applicable items) x 100]. Consistent with the original development paper,^9^ the present authors categorised quality as 76% to 100% (excellent), 51% to 75% (good; minor problems), 26% to 50% (major problems), and 0% to 25% (very major problems).Global Quality Scale (GQS):This uses a five-point Likert scale to rate overall quality and usefulness of health-related content (1 = very poor; 5 = excellent).Journal of the American Medical Association (JAMA) benchmark criteria: These were used to assess four transparency-related domains: authorship, attribution or references, currency or date, and disclosure. Each domain was scored as 1 if explicitly present and 0 if absent, yielding a total score ranging from 0 to 4 for each response. Because chatbot responses generally do not have conventional authorship or publication dates, these criteria were interpreted as indicators of transparency and source reporting rather than as direct measures of factual accuracy.^20^


### Raters and Adjudication

All evaluations using the instruments were conducted independently by two raters with more than 10 years of clinical experience in oral and maxillofacial surgery. Discrepancies were first addressed through discussion. If no consensus could be reached, a third senior expert acted as an adjudicator, and as such, the rating of that expert was final.

### Statistical Analysis

This study followed a non-parametric approach for statistical analysis. The present authors first evaluated normality using a Kolmogorov-Smirnov test, and the data for both reliability and readability were found to be non-normally distributed. Thus, a Kruskal- Wallis test was employed to evaluate overall differences among the LLMs, and in instances where statistically significant differences were found, a Dunn test was used for post hoc pairwise comparisons.

For readability, a readability score was calculated for each chatbot’s response following each of the pre-defined readability indices. Since these scores also did not meet normality, a Kruskal-Wallis test was again applied to compare the four LLMs for readability. To determine whether the readability level complied with a sixth-grade readability level, a Wilcoxon signed-rank test was conducted to compare the readability scores of each LLM to the previously established readability score at a sixth-grade level. Analyses were conducted in SPSS version 26.0 (IBM, Armonk, NY, USA), with the level of statistical significance set at *P* < 0.05.It is important to clarify that inter-rater reliability (e.g., kappa) was not determined because all assessment items had been agreed to ahead of time through discussion between two experts. The intraclass correlation coefficient (ICC) was used to assess consistency across evaluation tools.

### Reliability Analysis 

DISCERN, EQIP, the JAMA benchmarks, and GQS were used to summarize performance for each of the four AI systems (Table 2; mean ± standard deviation [SD]). Results of Kruskal-Wallis tests found statistically significant between-system differences for DISCERN (*P* < 0.001), EQIP (*P* < 0.001), and JAMA (*P* < 0.001). GQS analysis did not find a statistically significant difference between systems (*P* = 0.440).

**Table 2 Table2:** Reliability and quality scores across AI LLMs, mean ± SD

Mode	DISCERN	EQIP	GQS	JAMA
ChatGPT	38.09 ± 6.75	70.00 ± 0.00	3.77 ± 0.69	0.0 ± 0.0
DeepSeek	40.68 ± 6.22	71.36 ± 3.51	3.82 ± 0.73	0.0 ± 0.0
Gemini	38.55 ± 5.86	70.23 ± 1.07	3.77 ± 0.75	0.0 ± 0.0
Perplexity	46.36 ± 4.70	85.00 ± 0.00	4.05 ± 0.58	1.0 ± 0.0
H	20.148	74.354	2.702	87.000
*P* value	< 0.001	< 0.001	0.440	< 0.001


When assessing using DISCERN, scores of content quality by system revealed variation. Perplexity achieved the highest score (46.36), followed by DeepSeek (40.68). The scores of ChatGPT (38.09) and Gemini (38.55) were relatively low and comparable to each other (Fig 1). Data from EQIP indicated that all systems were in the ‘good (minor problems)’ range, with Perplexity statistically higher (85.00) than ChatGPT (70.00), DeepSeek (71.36), and Gemini (70.23; Fig 1). Overall quality for GQS demonstrated that content quality was above average, with Perplexity at 4.05, DeepSeek at 3.82, Gemini at 3.77, and ChatGPT at 3.77, suggesting the systems provided appropriate flow of information and usability, yet could be improved by depth of information and domain specificity (Fig 1). Using JAMA benchmark criteria, Perplexity had a higher mean JAMA score (Fig 1).

**Fig 1 Fig1:**
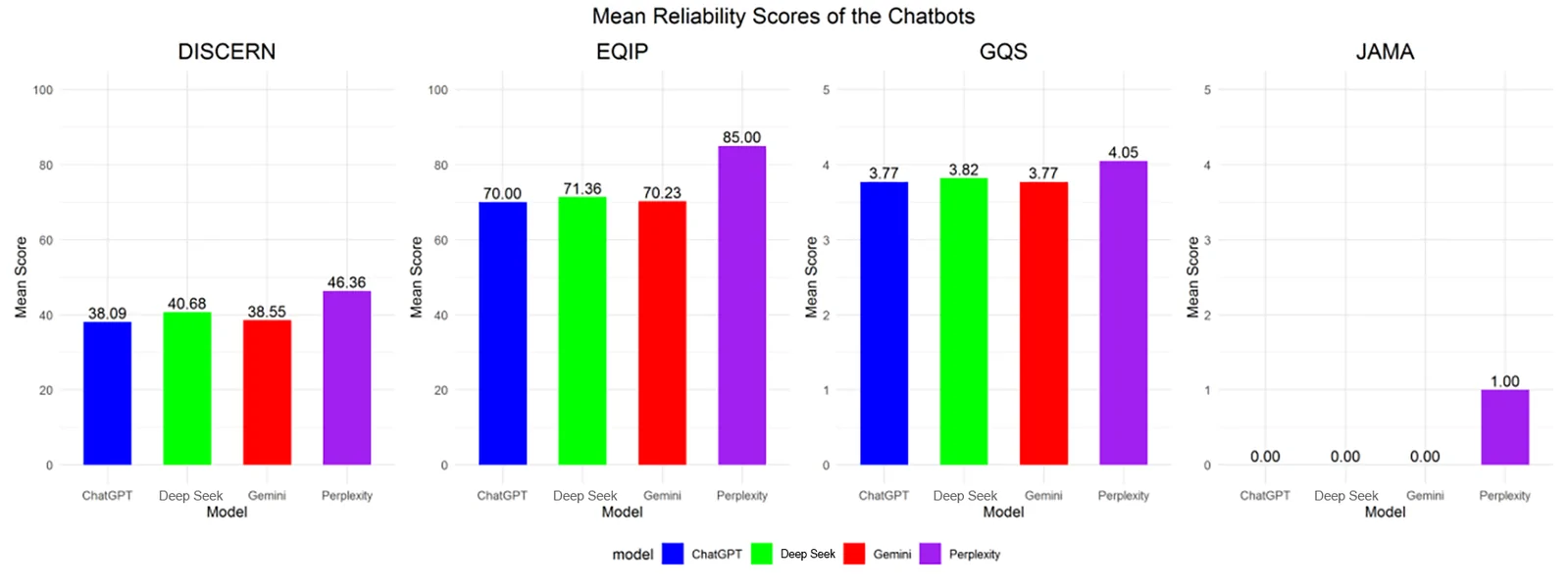
Mean reliability and quality scores of the LLMs based on DISCERN, EQIP, GQS, and JAMA.

### Post Hoc Pairwise Comparisons 

Results from the Dunn multiple-comparison test indicated systematic differences (Table 3). In terms of content quality (DISCERN, EQIP), Perplexity was statistically higher in comparison to ChatGPT (DISCERN, *P* = 0.0006; EQIP, *P* < 0.001), Gemini (DISCERN, *P* = 0.0004; EQIP, *P* < 0.001), and DeepSeek (DISCERN, *P* = 0.01; EQIP, *P* < 0.001). No significant differences were seen among ChatGPT, DeepSeek, or Gemini for these quality metrics (*P* > 0.05). With regard to publishing standards (JAMA), Perplexity was statistically significantly different from ChatGPT, DeepSeek, and Gemini (*P* < 0.001). For overall quality (GQS), no pairwise comparisons reached statistical significance (*P* > 0.05), which indicates that users rated user experience similarly across models. Overall, Perplexity showed relatively higher scores on DISCERN, EQIP, and JAMA, suggesting better performance in certain aspects of information structure, transparency, and source-related reporting. However, GQS scores did not differ significantly among models, and DISCERN scores across all models remained within modest quality ranges. Therefore, these findings should be interpreted as metric-specific relative differences rather than evidence of broad clinical superiority of any single chatbot.

**Table 3 Table3:** Dunn post hoc pairwise comparisons for reliability and quality scores, *P* values

Pairwise comparison	DISCERN	EQIP	JAMA	GQS
ChatGPT/DeepSeek	0.4056	0.4398	1.0	1.0
ChatGPT/Gemini	0.9529	0.8118	1.0	0.9664
DeepSeek/Gemini	0.3563	0.4994	1.0	1.0
ChatGPT/Perplexity	0.0006	0.0	0.0	0.5055
DeepSeek/Perplexity	0.01	0.0	0.0	0.4739
Gemini/Perplexity	0.0004	0.0	0.0	0.9351


### Readability Evaluation

The six widely used readability indices, namely ARI, FRES, FOG, FKGL, CL, and SMOG, were evaluated (Table 4). As shown in Fig 2, no chatbot met the sixth-grade benchmark across indices. FRES scores were well below the recommended eighth grade (80 to 90) for readability. FOG and FKGL also indicated readability at a grade level above the point for easily understandable text for the public.

**Table 4 Table4:** Readability scores across AI LLMs, mean ± SD

Program	ARI	CL	FKGL	FOG	FRES	SMOG
ChatGPT	14.36 ± 3.39	13.62 ± 2.48	12.65 ± 3.07	12.99 ± 2.78	39.86 ± 15.21	10.89 ± 2.22
DeepSeek	10.84 ± 1.74	11.82 ± 1.56	13.65 ± 1.60	11.39 ± 1.41	49.95 ± 8.96	9.57 ± 1.05
Gemini	11.09 ± 2.59	11.14 ± 2.09	14.65 ± 2.28	11.10 ± 2.09	52.59 ± 12.47	9.56 ± 1.71
Perplexity	13.87 ± 4.33	14.08 ± 3.27	15.65 ± 3.36	13.30 ± 2.46	37.50 ± 14.27	11.00 ± 2.35
Sixth-grade level score	6	6	6	6	80–90	6


**Fig 2 Fig2:**
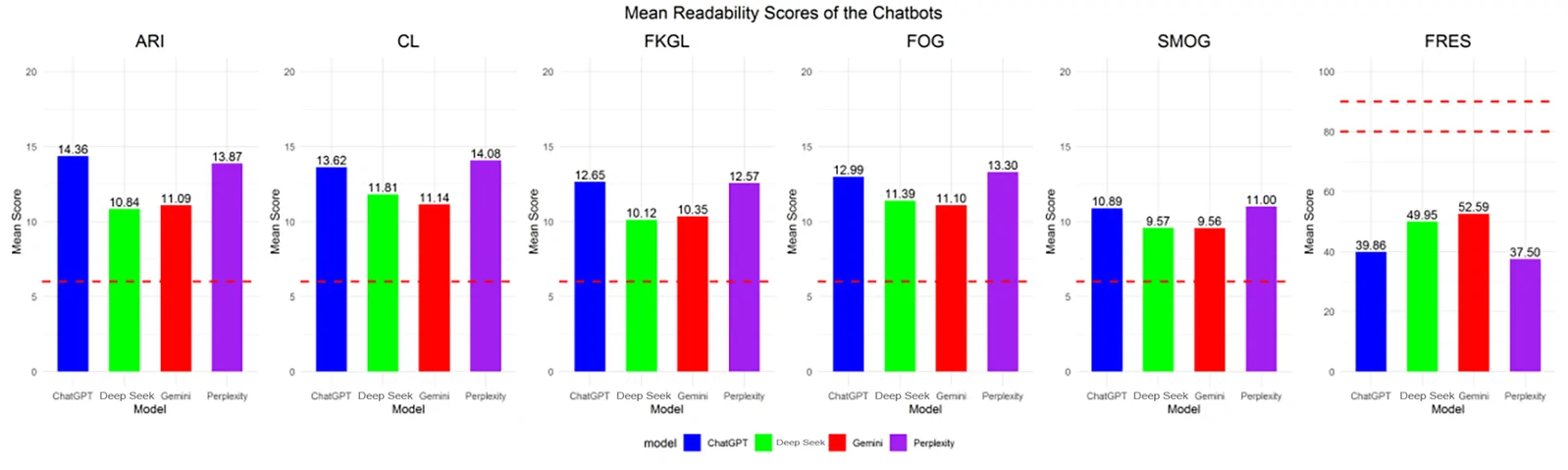
Mean readability scores of the LLMs based on ARI, FOG, FKGL, CL, SMOG, and FRES. The red reference line indicates the recommended sixth-grade readability benchmark for patient education materials, except for FRES, for which higher scores indicate easier readability.

Kruskal-Wallis tests were used to compare readability scores among the four LLMs. To assess whether the readability scores met the predefined sixth-grade benchmark, one-sample Wilcoxon signed-rank tests were conducted for each chatbot against the corresponding benchmark value. The results indicated that the initial responses generated by all four LLMs exceeded the recommended readability level for patient education materials.

A heatmap showing the correlations among readability indices is presented in Fig 3. The colour gradient from blue to red reflects the increasing scores for different readability indices and provides a visual summary of which indices are more aligned than others and in which areas they diverge.

**Fig 3a and b Fig3aandb:**
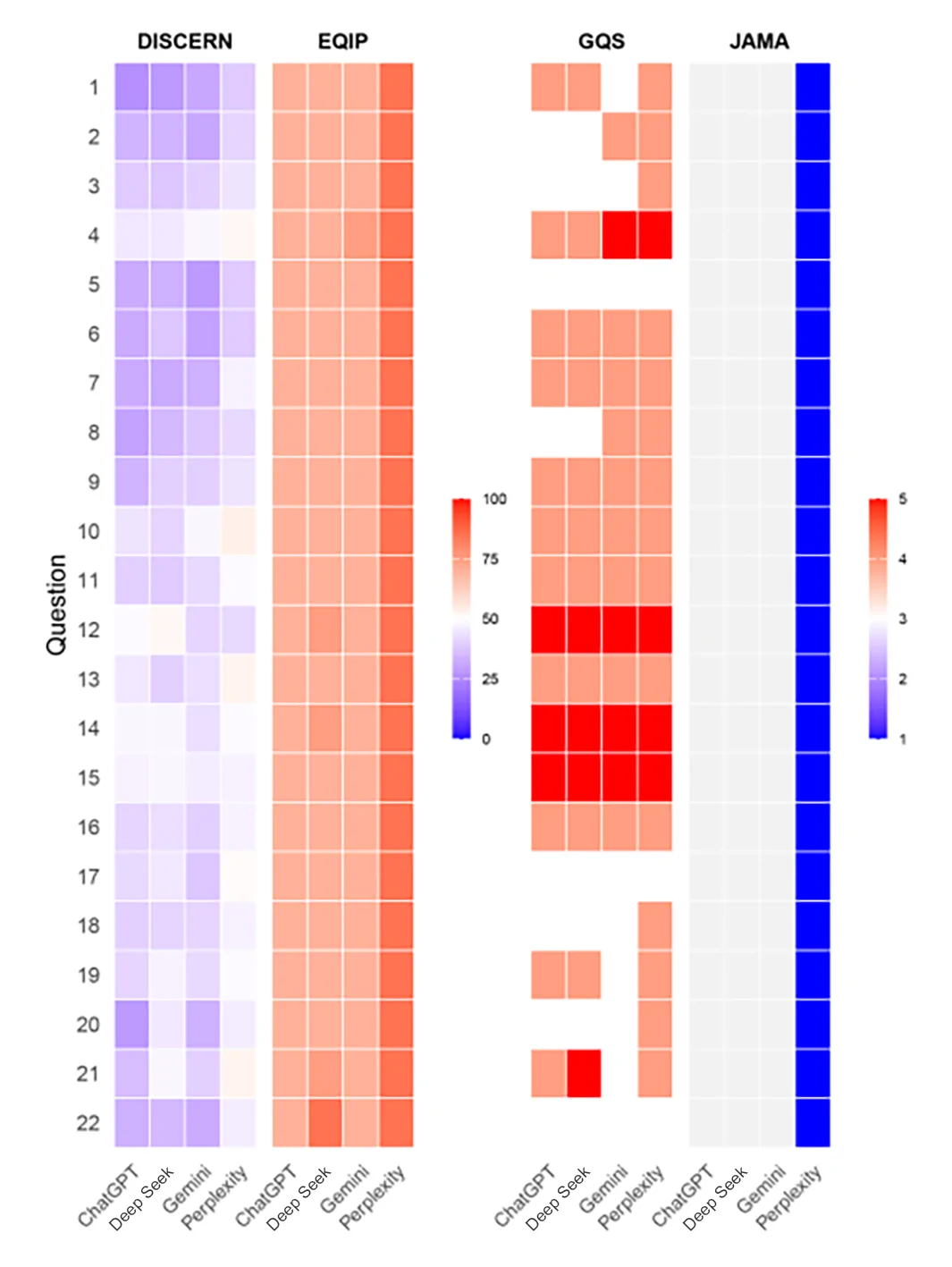

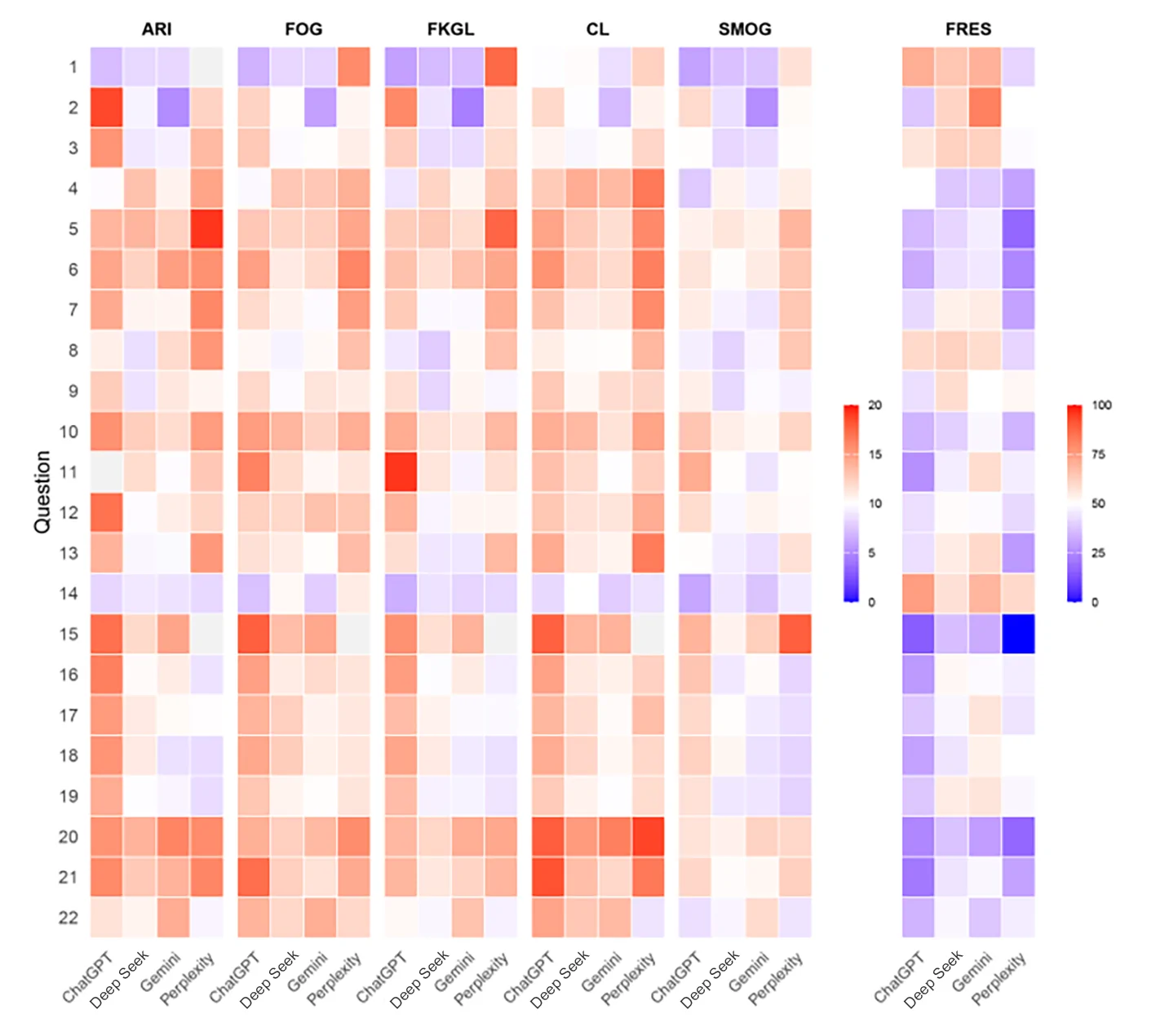
Heatmap of reliability (a) and readability scores (b) across chatbot responses.

## Discussion

Accurately evaluating the informational quality and public understandability of outputs is becoming more significant as these systems are increasingly applied in potentially sensitive health consultation scenarios. As the first multidimensional assessment of the informational quality and public comprehensibility of LLMs outputs for oral cancer, this study synthesises the present authors’ findings with existing evidence to demonstrate that current LLMs still fail to provide health information that is both high-quality and easily understandable.

### Quality of AI-generated Health Information

Four complementary measures, namely DISCERN, EQIP, GQS, and the JAMA benchmarks, were employed to measure the quality of chatbot outputs. Each measure captures different but sufficiently overlapping dimensions. DISCERN and EQIP, as structured schemes to measure consumer health information, both exhibit good reliability; prior work shows better inter-rater reliability for DISCERN,^15^ which focuses on clarity, balance, and completeness of information, whereas EQIP provides a finer-grained measurement from a clinical practice perspective.

In contrast to these structured measures, GQS provides a quick, global, five-point measure of the total quality and usefulness of the output. The JAMA benchmarks bring in research rigour as part of the measures, focusing on measures for authorship, attribution/references, currency, and disclosure.

It should be noted that these measures were originally developed to apply to static written material and may not capture the pacing and dialogic, dynamically generated aspects of chatbot responses. Nevertheless, there are significant correlations among the measures reported,^8^ and the present authors support the application of a multi-measure approach to measure reliability and quality. Perplexity scored better than DeepSeek, ChatGPT, and Gemini on multiple measures of information trustworthiness, especially DISCERN and EQIP, demonstrating the value of tracking information sources when examining health-related policy. By giving reference to supporting research around the information or making efforts to establish its provenance, the text and presentation can appear more authoritative. Similarly, Gemini scored lower on DISCERN and JAMA, which may be linked to the inability for users to provide a reference in a longitudinal fashion or establish provenance from their interaction history, thus eliminating the inherent citation element.^5^ Since the source provenance or authorship was elevated when designing the model, it would be expected that the user will have difficulty assessing the authority of this information, which erodes trustworthiness in health-related information.

Transparency of information is a fundamental principle in trust in health communication. As the public increasingly relies on conversational systems for answers to their medical questions, unsupported claims become more dangerous, and dissemination of faulty information could make the situation worse. Additionally, the risk of unsupported information becomes more acute when users are discussing sensitive health topics where inappropriate or inaccurate information may lead patients to delay care, result in a wider community spread of misinformation, or have even larger public health ramifications around other matters (e.g. sexually transmitted infections). The above results regarding the spread of unsubstantiated medical claims should be interpreted within the methodological boundaries of conventional health information appraisal tools, which are further discussed in the Limitations section.

### Challenges of Readability

Readability was a major focus of this study because the usefulness of AI-generated health information depends not only on information quality but also on whether lay users can understand and apply the content. Although FRES and FKGL are widely used readability indices, previous empirical work suggests that SMOG may be particularly relevant for medical texts, especially when a higher standard of comprehension is considered.^23^


In this study, all evaluated LLMs generated responses above the recommended sixth-grade reading level for patient education materials. In the context of oral cancer, linguistically complex explanations may reduce patients’ ability to recognise warning signs, understand risk factors, prepare for diagnostic procedures, and follow recommendations for treatment or follow-up. This concern is particularly relevant for individuals with limited health literacy, older adults, and users who search for cancer-related symptoms while experiencing anxiety or uncertainty.

However, these findings should be interpreted cautiously. Readability formulas mainly assess lexical and syntactic complexity and do not directly measure actual patient comprehension, emotional response, trust formation, or decision making. In addition, conversational AI systems may be able to simplify explanations during follow-up interactions, which was not captured by the single-turn design of this study. Therefore, the present results indicate that the initial chatbot responses were linguistically demanding, rather than proving that users would necessarily fail to understand them. Future studies should combine formula-based readability analysis with user testing, comprehension assessment, and evaluation of readability adaptation in multi-turn conversations.

### Limitations

This study has several limitations. First, the 22 oral cancer–related prompts were derived from Google Trends and expert consultation. Although this approach balanced public interest with clinical relevance, the question set could not capture all patient information needs, and formal qualitative saturation was not assessed. Google Trends also lacks demographic information and conventional sample size data; therefore, search behaviour may be influenced by internet access, language, culture, and health literacy.^1,14,29
^ Second, only single-turn responses were evaluated. In real-world use, patients may ask follow-up questions, request simpler explanations, or provide additional clinical details, which could change the reliability, readability, and communication quality of LLM-generated responses. Third, the assessment tools were originally developed mainly for static written health information. DISCERN, EQIP, GQS, and JAMA assess information quality, transparency, and usefulness, but they do not directly measure factual accuracy, contextual appropriateness, hallucination risk, or clinical safety. Similarly, readability formulas estimate textual complexity rather than actual patient comprehension or decision making. Finally, the findings may be affected by differences in model architecture, citation functions, access modes, platform settings, and rapid model updates. The free-access version of ChatGPT was not evaluated separately because the models and access modes had been prespecified before data collection; adding a new model afterwards would have introduced post hoc heterogeneity in model version, access mode, and query timing. Future studies should include larger question sets, free and subscription-based LLM versions, multi-turn interactions, guideline-based accuracy assessment, hallucination detection, and user-centred comprehension testing.

### Practical Implications

The present findings have practical implications for patients, clinicians, developers, and regulators. For patients, AI LLMs may provide convenient access to oral cancer–related information, but the current outputs should not be regarded as a substitute for professional consultation. For clinicians, it may be useful to recognise that patients increasingly encounter AI-generated information and may need guidance in interpreting uncertain, incomplete, or overly complex content. For developers, the results highlight the need to improve source attribution, uncertainty communication, risk warnings, and plain-language adaptation. For regulators and professional organisations, cancer-related AI health information may require clearer standards for transparency, accountability, and safety monitoring.

Importantly, the relatively higher scores achieved by Perplexity for some reliability-related metrics should not be interpreted as evidence of clinical accuracy or independent clinical usefulness. Rather, these suggest that source-related transparency and structured presentation may improve instrument-based information quality. Future systems should combine verifiable sourcing with guideline-based accuracy checks, hallucination detection, and health literacy–sensitive language generation.

### Future Perspectives

Future research should move beyond passive evaluation of general-purpose LLMs and explore the development of customised, guideline-linked LLM systems for oral cancer patient education. Such systems should incorporate curated clinical knowledge bases, verifiable citations, hallucination detection, risk warnings, and health literacy–sensitive rewriting functions. In parallel, patient-facing educational materials should be developed to explain the potential benefits and limitations of LLM-generated oral cancer information, helping patients use these tools as supplementary information sources rather than substitutes for professional consultation.

### Formation of a Vital Analysis Paradigm

Future evaluation frameworks should shift from static single-turn assessment to interactive evaluation systems capable of measuring multi-turn coherence, contextual consistency, error correction, and subsequent follow-up processing. Rather than relying on one-off snapshot evaluations, longitudinal tracking across various LLMs is encouraged to explore how version updates affect the accuracy, timeliness, and conversational features of generated replies. Furthermore, evaluation work can evolve from subjective manual scoring towards objective automatic workflows, in which LLMs serve as auxiliary tools for preliminary fact-checking and consistency verification, to streamline operational steps and improve the overall scalability and efficiency of quality appraisal.

### Marketing of Technical Standardisation

To establish unified performance standards for content generation, cross-disciplinary cooperation among clinicians, information scientists and ethicists is essential to formulate industrial specifications. These standards require content strictly based on clinical guidelines and explicit source citations such as accessible links or DOIs to guarantee credibility. Meanwhile, developers can build embedded structured citation frameworks and real-time fact-checking mechanisms as core trust-building modules, which can verify key medical statements and effectively decrease hallucinations during content generation. In addition, standardised risk prompts and disclaimers need to be formulated to clarify accountability and usage boundaries, requiring LLMs to actively remind users to seek professional medical advice when involving symptom description and treatment decision making.

### Making Knowledge Delivery More Effective

To boost the efficiency of medical knowledge transmission, LLMs can be tightly connected to rigorously curated knowledge resources including specialised databases and institutional knowledge graphs to greatly elevate the accuracy and specificity of generated answers. Targeted readability optimisation algorithms can also be developed to replace rigid scoring rules for medical texts, while automatic syntactic simplification can break long complicated sentences into concise expressions, and professional jargon can be automatically identified and converted into plain explanatory language for ordinary users. Moreover, tiered explanatory modes can be designed to adaptively output content of varying complexity from simple popular science versions to detailed professional introductions according to dialogue contexts and user groups. Collectively, unified dynamic quantification assessment, standardised technical specifications, and user-orientated content optimisation constitute a feasible roadmap to transform medical AI into an accurate, secure, and interpretable trusted information service.

## Conclusion

This study provides a multidimensional evaluation of LLM responses to oral cancer–related questions, focusing on instrument-based reliability, information quality, transparency, and readability. Significant differences were observed among LLMs in DISCERN, EQIP, and JAMA scores, whereas no significant difference was found in GQS scores. Perplexity recorded relatively higher scores on several reliability-related metrics; however, these findings should be interpreted as metric-specific relative differences rather than evidence of overall clinical superiority.

None of the evaluated LLMs produced responses that met the recommended sixth-grade readability level for patient education materials, indicating that their initial outputs may be linguistically demanding for lay users. Overall, AI LLMs may have adjunctive value in oral cancer information provision, but their current use remains limited by variable information quality, insufficient transparency, and poor readability. Future development and evaluation should emphasise verifiable sourcing, guideline-based factual accuracy assessment, hallucination detection, risk communication, multi-turn interaction testing, and plain-language adaptation to support safer and more accessible patient education.

## References
